# Hepatocyte-like cells derived from human induced pluripotent stem cells using small molecules: implications of a transcriptomic study

**DOI:** 10.1186/s13287-020-01914-1

**Published:** 2020-09-11

**Authors:** Xiugong Gao, Rong Li, Patrick Cahan, Yang Zhao, Jeffrey J. Yourick, Robert L. Sprando

**Affiliations:** 1 Division of Toxicology, Office of Applied Research and Safety Assessment, Center for Food Safety and Applied Nutrition, U.S. Food and Drug Administration, Laurel, MD 20708 USA; 2 Department of Biomedical Engineering, Institute for Cell Engineering, Johns Hopkins University School of Medicine, Baltimore, MD 21205 USA

**Keywords:** Hepatocyte differentiation, Hepatocyte-like cells, Induced pluripotent stem cells, Transcriptomics, Small molecules, Microarray

## Abstract

**Background:**

Hepatocyte-like cells (HLCs) derived from human induced pluripotent stem cells (iPSCs) hold great promise in toxicological applications as well as in regenerative medicine. Previous efforts on hepatocyte differentiation have mostly relied on the use of growth factors (GFs) to recapitulate developmental signals under in vitro conditions. Recently, the use of small molecules (SMs) has emerged as an attractive tool to induce cell fate transition due to its superiority in terms of both quality and cost. However, HLCs derived using SMs have not been well characterized, especially on the transcriptome level.

**Methods:**

HLCs were differentiated from human iPSCs using a protocol that only involves SMs and characterized by transcriptomic analysis using whole genome microarrays.

**Results:**

HLCs derived using the SM protocol (HLC_SM) displayed specific hepatic marker expression and demonstrated key hepatic functions. Transcriptomic analysis of the SM-driven differentiation defined a hepatocyte differentiation track and characterized the expression of some key marker genes in major stages of hepatocyte differentiation. In addition, HLC_SM were scored with CellNet, a bioinformatics tool quantifying how closely engineered cell populations resemble their target cell type, and compared to primary human hepatocytes (PHHs), adult liver tissue, fetal liver tissue, HLCs differentiated using GFs (HLC_GF), and commercially available HLCs. Similar to HLC_GF, HLC_SM displayed a mixed phenotype of fetal and adult hepatocytes and had relatively low expression of metabolic enzymes, transporters, and nuclear receptors compared to PHHs. Finally, the differentially expressed genes in HLC_SM compared to HLC_GF and to PHHs were analyzed to identify pathways and upstream transcription regulators which could potentially be manipulated to improve the differentiation of HLCs.

**Conclusions:**

Overall, the present study demonstrated the usefulness of the SM-based hepatocyte differentiation method, offered new insights into the molecular basis of hepatogenesis and associated gene regulation, and suggested ways for further improvements in hepatocyte differentiation in order to obtain more mature HLCs that could be used in toxicological studies.

## Background

In recent years, there has been burgeoning interest in hepatocyte-like cells (HLCs) derived from human induced pluripotent stem cells (iPSCs). In addition to their therapeutic value in regenerative medicine such as hepatocyte transplantation for intractable liver diseases [1], these cells also hold great promise in disease modeling, mechanistic study, drug development, and toxicological applications [2–4]. Compared to primary human hepatocytes (PHHs), which are considered the gold standard for predictive in vitro models in pharmacology and toxicology [5], HLCs afford the advantages of unlimited supply, consistency in quality, and sustainability of function in long-term culture. More importantly, since iPSCs could be derived from different donors, therefore, for toxicological applications, iPSC-derived HLCs are uniquely suited for studying idiosyncratic drug-induced liver injury (iDILI) [6] and for toxicity testing and risk assessment on susceptible subpopulations [7, 8].

Previous efforts on hepatocyte differentiation have mostly relied on the use of growth factors (GFs) to recapitulate developmental signals under in vitro conditions, such as activin A, bone morphogenetic protein 4 (BMP4), fibroblast growth factor 2 (FGF2), hepatocyte growth factor (HGF), and oncostatin M [9]. Cells generated using such protocols showed encouraging results in terms of marker expression and functional assays, but in all cases displayed an immature hepatic phenotype that more closely resembles fetal rather than adult hepatocytes [10]. Moreover, the use of GFs in these protocols has the disadvantages of high cost, low reproducibility, and potential safety concerns for clinical applications.

Recently, increasing studies have shown the feasibility and advantages of using small molecules (SMs) to induce cell fate conversion [11]. A protocol was published by Siller et al. [12] to differentiate iPSCs into HLCs that only involves SMs. CHIR99021, a glycogen synthase kinase (GSK)-3 inhibitor, was used for definitive endoderm (DE) induction, dimethyl sulfoxide (DMSO) was used for hepatic specification, and N-hexanoic-Tyr, Ile-(6) amino-hexanoic amide (dihexa, an HGF receptor agonist), and dexamethasone were used for hepatocyte maturation. The resultant HLCs reportedly demonstrate similar levels of function to those derived using previous approaches based on GFs.

In the current study, to gain a more comprehensive and unbiased assessment of the HLCs differentiated using SMs (HLC_SM), we conducted a whole genome microarray study and characterized gene expression changes during the course of the SM-driven differentiation and assessed the expression of some key marker genes in major stages of hepatocyte differentiation. In addition, global gene expression of the HLC_SM was compared to PHHs, adult liver tissue, fetal liver tissue, HLCs differentiated using GFs (HLC_GF), and commercially available HLCs. Furthermore, transcription regulators that may be responsible for the differential gene expression between HLCs and PHHs were identified which could potentially be manipulated to improve the differentiation of HLCs.

## Methods

### Cell culture

Human iPSCs (S06) were identified and characterized as previously reported [13]. Cells were maintained as single-cell culture at 37 °C, 5% CO_2_, and > 90% humidity in Cellartis DEF-CS Culture System (Takara Bio USA, Mountain View, CA). The culture medium was changed every day until the cells reach a confluence of 1.5–3.0 × 10^5^ cells/cm^2^, which normally occurred 3–4 days post passage. With each passage, the cells were re-dissociated into single cells by TrypLE Select 1× (Gibco, Gaithersburg, MD) and transferred to a new tissue culture plate coated with COAT-1 (Takara Bio USA) at a density of 4.0–5.0 × 10^4^ cells/cm^2^.

iCell Hepatocytes 2.0 were purchased from Fujifilm Cellular Dynamics, Inc. (FCDI; Madison, WI) and cultured following the manufacturer’s protocols. Briefly, cells were thawed and seeded onto rat tail collagen type I-coated surfaces (3 × 10^5^ cells/cm^2^) using the plating medium composed of RPMI medium, B27 supplement, oncostatin M (20 ng/ml), dexamethasone (0.1 μM), gentamicin (25 μg/ml), and the proprietary iCell Hepatocytes 2.0 Medium Supplement. Plating medium was replenished daily for 4 days after seeding. On day 5, the plating medium was replaced by the maintenance medium, which was composed of RPMI medium, B27 supplement, dexamethasone (0.1 μM), gentamicin (25 μg/ml), and the medium supplement. Maintenance medium was refreshed every 2 days. Cell culture media and medium supplements (RPMI medium, B27 supplement, dexamethasone, gentamicin, and rat tail collagen type I) were all obtained from Thermo Fisher Scientific (Waltham, MA) except for oncostatin M, which was obtained from R&D Systems (Minneapolis, MN).

PHHs were obtained from Thermo Fisher Scientific (Waltham, MA). Each sample was a pooled population of 10 donors including both males and females. Cells were thawed, washed, and used for microarray studies without any treatment.

### Hepatocyte differentiation by only SMs

SM-driven hepatocyte differentiation was carried out following a published protocol [12] with slight modifications. In brief, cells were seeded onto 6-well plates coated with COAT-1 (Takara Bio USA) at a density of 5.0 × 10^4^ cells/cm^2^ and allowed to adhere for 24 h in DEF-CS medium (Takara Bio USA) at 37 °C, 5% CO_2_. Cells became near confluent the next day. The differentiation comprises of three phases. Phase I (DE induction) consists of a 24-h treatment with 3 μM CHIR99021 (Stemgent, Cambridge, MA) in RPMI-B27 (RPMI 1640 + B27 supplement/B27 supplement minus insulin (mixed in 1:3 ratio), all from Thermo Fisher Scientific), followed by culture in RPMI-B27 alone (without CHIR99021) for another 24 h. Massive cell death was observed during phase I, with only 30–40% cells survived. Phase II (hepatic specification) consists of 5-day treatment with 1% DMSO (Sigma-Aldrich, St. Louis, MO) in DMEM containing 20% knockout serum replacement, 2 mM GlutaMAX, 1× MEM non-essential amino acids (all from Thermo Fisher Scientific), and 100 μM 2-mercaptoethanol (Sigma-Aldrich). Phase III (hepatocyte maturation) consists of 10-day treatment with 100 nM dihexa (MedChemExpress, Monmouth Junction, NJ) and 100 nM dexamethasone (Sigma-Aldrich) in Leibovitz L-15 media containing 8.3% tryptose phosphate broth, 10 μM hydrocortisone 21-hemisuccinate, 50 μg/ml sodium-l-ascorbate (all from Sigma-Aldrich), 0.58% insulin-transferrin-selenium (ITS), 2 mM GlutaMAX (both from Thermo Fisher Scientific), and 8.3% fetal bovine serum (Lonza). During phases II and III, the medium was replenished every 2 days. Phase III was extended for 7 more days to test if the maturation of the cells improves.

### Hepatocyte differentiation using GFs

The Cellartis iPS Cell to Hepatocyte Differentiation System (Takara Bio USA) was used for comparison with the SM-driven protocol. According to the manufacturer, this differentiation system contains GFs [14]. To carry out the differentiation, instructions from the manufacturer were followed. First, iPSCs were guided to differentiate into DE using the Cellartis Definitive Endoderm Differentiation Kit (Takara Bio USA). Briefly, on day 0, cell culture vessels were coated using the COAT-1 coating solution (Takara Bio USA). iPSCs were dissociated into single cells, re-suspended in warm DE diff day 0 medium and seeded with a density of 3.0–4.0 × 10^3^ cells/cm^2^ (in 0.2 ml medium/cm^2^). On days 1, 2, 3, 4, and 6, medium changes were performed using warm DE diff medium designated for the specific day. On day 7, the DE cells were enzymatically dissociated and further differentiated using the Cellartis Hepatocyte Differentiation Kit (Takara Bio USA). In brief, DE cells were seeded in the hepatocyte thawing and seeding medium at an initial density of 1.2–1.3 × 10^5^ cells/cm^2^ in 24-well plate format in 1 ml of medium. The DE cells were differentiated in the hepatocyte thawing and seeding medium for 2 days at 37 °C, before changing to the hepatocyte progenitor medium for another 5 days of differentiation to hepatoblasts. The cells were then further differentiated in the hepatocyte maturation medium for 4 days and finally matured in the hepatocyte maintenance medium for another 14 days. The medium was exchanged every 2–3 days following the manufacturer’s instructions.

### Immunofluorescence staining

Cells were fixed in 4% (v/v) paraformaldehyde (Alfa Aesar; Tewksbury, MA) for 15 min at room temperature. After washing with PBS containing 0.2% (v/v) Tween (PBST) (Fisher Scientific; Waltham, MA) for three times, cells were then permeabilized by 0.15% (v/v) TritonX-100 (Sigma-Aldrich; St. Louis, MO) in PBS for 1 h at 25 °C. After permeabilization, cells were blocked with 1% (v/v) bovine serum albumin (BSA) (Invitrogen; Carlsbad, CA) in PBST (PBSTB) for 30 min at 25 °C. After gentle removal of PBSTB, cells were incubated with primary antibodies conjugated to Alexa Fluor 488, 594, or 647 in PBSTB overnight at 4 °C. Antibodies against C-X-C chemokine receptor type 4 (CXCR4), SRY (sex-determining region Y)-box 17 (SOX17), alpha-fetoprotein (AFP), and albumin (ALB) were from R&D Systems, and antibodies against hepatocyte nuclear factor 4 alpha (HNF4A) and alpha-1-antitrypsin (A1AT) were from Abcam. Subsequently, cells were washed three times in PBST and stained with Hoechst dye (Invitrogen). Images of the stained cells were taken under an EVOS FL imaging system (Thermo Fisher Scientific). The expression rate of stage-specific markers and the mean fluorescence intensity of marker expression in the cells were calculated from immunofluorescence staining images using the ImageJ software [15]. Three random images for each marker were used in the calculation, and approximately 900 cells in total were counted per marker.

### Protein extraction and quantification

Cells were lysed in Pierce RIPA buffer (Thermo Fisher Scientific) supplemented with 1× Halt Protease Inhibitor Cocktail (Thermo Fisher Scientific) for 5 min at room temperature. The supernatant was collected after centrifugation at 1600*g* for 10 min, and total protein was quantified using Pierce BCA Protein Assay kit (Thermo Fisher Scientific) following the instructions of the manufacturer.

### Albumin and fibronectin secretion assay

HLCs were incubated in 1 ml of hepatocyte maintenance medium for 24 h, and the cell culture supernatant was collected and stored at − 80 °C until use. Enzyme-linked immunosorbent assay (ELISA) kits from Abcam were used to determine ALB and fibronectin level in the cell culture supernatant following the manufacturer’s instructions. Wells without cells were included as blank control. Results were normalized to protein quantity and represented the average of four independent experiments; error bars represent standard deviation.

### Urea synthesis

HLCs were treated with different concentrations of NH_4_Cl (0, 2, and 5 mM) in 1 ml of fresh medium for 24 h. Cell culture supernatant were then collected and analyzed for urea production using the Urea Assay Kit from Abcam following the instructions of the manufacturer. Wells without seeded cells were included as blank control. Results were normalized to protein quantities and represented the average of four independent experiments; error bars represent standard deviation.

### Periodic acid-Schiff (PAS) staining

Cells were fixed in 4% (v/v) paraformaldehyde (Alfa Aesar), and glycogen storage was visualized by PAS staining using a kit from Sigma-Aldrich following the manufacturer’s instructions. Images were taken by a BZ-X810 Fluorescence Microscope from Keyence (Itasca, IL) using phase contrast lenses.

### CYP activity assay

CYP activities of HLCs were analyzed at the indicated day of differentiation following a protocol adapted from Asplund et al. [14]. Briefly, cells were carefully washed twice with pre-warmed Williams’ medium E (Phenol-red free, + 0.1% penicillin/streptomycin (PEST)). The assay was started by adding pre-warmed Williams’ medium E (phenol-red free) containing 0.1% PEST, 25 mM HEPES, 2 mM l-glutamine, and a probe substrate cocktail [14] at 130 μl/cm^2^ culture area. After incubation at 37 °C for 2 h, the assay was ended and 100 μl of the supernatant was collected and stored at − 80 °C until further analysis. LC/MS analysis was used to measure the formation of the specific metabolites for 6 CYP variants: acetaminophen (CYP1A), OH-bupropion (CYP2B6), 4-OH-diclofenac (CYP2C9), 4-OH-mephenytoin (CYP2C19), OH-bufuralol (CYP2D6), and 1-OH-midazolam (CYP3A). The metabolite concentrations were normalized to the amount of protein per well and the assay duration (2 h). A twofold serial dilutions of a metabolite cocktail (with known concentrations of all metabolites) with Williams’ medium E were analyzed the same day along with the samples to establish a standard curve for each metabolite.

### RNA extraction and quality assurance

Cultured cells were harvested and stored at − 80 °C before RNA extraction. Cells were lysed in RLT buffer (Qiagen, Valencia, CA) and homogenized using QIAshredder (Qiagen). Cell lysates were extracted for total RNA using EZ1 RNA Cell Mini Kit (Qiagen) on EZ1 Advanced XL automated RNA purification instrument (Qiagen) following the manufacturer’s protocol, including an on-column DNase digestion. Total RNA concentration and purity were subsequently measured using a NanoDrop 2000 UV-Vis spectrophotometer (NanoDrop Products, Wilmington, DE). RNA integrity was further analyzed by an Agilent 2100 Bioanalyzer (Agilent, Santa Clara, CA) using the RNA 6000 Nano Reagent Kit from the same manufacturer.

### Microarray experiment

All reagents and instruments used for the microarray experiment were obtained from Affymetrix (Santa Clara, CA). Total RNA from different cell samples was processed for gene expression profiling on GeneChip PrimeView Human Gene Expression Arrays using GeneChip 3′ IVT PLUS Reagent Kit following the manufacturer’s protocol. Briefly, 100 ng total RNA was used to generate single-stranded complementary DNA (cDNA) using reverse transcriptase and a T7-linked oligo (dT) primer, which was then converted to double-stranded cDNA using DNA polymerase and RNase H. The second strand of the double-stranded cDNA served as a template for the subsequent in vitro transcription (IVT) to synthesize the complementary RNA (cRNA) with biotinylated UTP and CTP using T7 RNA polymerase. The labeled cRNA was then purified and measured for concentration.

The purified cRNA (12 μg) was fragmented by divalent cations (Mg^2+^) at an elevated temperature (94 °C). Fragmented and labeled cRNA was hybridized to the GeneChip PrimeView Human Gene Expression Arrays at 45 °C for 16 h in the GeneChip Hybridization Oven 645. After hybridization, the array chips were stained and washed using the GeneChip Fluidics Station 450. Finally, the chips were scanned using GeneChip Scanner 3000 7G. The scanned image (DAT) files were preprocessed using Affymetrix GeneChip Command Console software 4.0 to produce cell intensity (CEL) files. All arrays were assessed for data quality using Affymetrix Expression Console software 1.3 prior to further data analysis.

### Microarray data analysis

The values of individual probes belonging to one probe set in the CEL files were summarized using the robust multi-array average (RMA) algorithm [16] embedded in the Affymetrix Expression Console software 1.3. Principal component analysis (PCA) and hierarchical clustering analysis (HCA) were conducted using ArrayTrack developed by U.S. Food and Drug Administration [17].

For the selection of differentially expressed genes (DEGs), statistical analysis between two experimental groups was conducted using Affymetrix Transcriptome Analysis Console software 2.0, based on one-way analysis of variance (ANOVA) and Welch *t*-test. For each comparison, the fold change (FC) of every annotated gene, together with their ANOVA *p* value or false discovery rate (FDR), was used for the selection of DEGs with cutoff values described in the text.

### Pathway analysis and upstream regulator analysis

Pathway analysis was conducted using the online tool Database for Annotation, Visualization, and Integrated Discovery (DAVID) (https://david.ncifcrf.gov/) to identify KEGG pathways overrepresented by the DEGs. The whole genome of *Homo sapiens* (human) was used as the background, and enrichment was statistically determined using default settings in DAVID. Upstream analysis was carried out using the Ingenuity Pathway Analysis (IPA) software with default settings to identify various upstream regulators of the DEGs including transcription regulators.

### Reference datasets

Searching the GEO database identified three entries (GSM3236134 [18], GSM1633257, and GSM1633258) describing microarray data of HLCs on the Affymetrix GeneChip PrimeView Human Gene Expression Array platform. These datasets were included in the analysis for comparison and were renamed as “HLC_Wruck,” “HLC_Yang_1,” and “HLC_Yang_2,” respectively.

### Cell and tissue type classification using CellNet

Cell and tissue (C/T) classification was performed using the CellNet platform as described previously [19]. C/T classifiers for the array type used in this study (GeneChip PrimeView Human Gene Expression Array) were trained using training data sets obtained on the Human Genome U133 Plus 2.0 Array platform.

### Statistical analysis

Student’s *t*-test was used to analyze statistical significance of differences between different groups in functional analysis of HLCs. The mean, standard deviation (SD), and *p* values were calculated using the Microsoft Excel software.

## Results

### Differentiation of iPSCs to HLCs driven by SMs

**Fig. 1:**
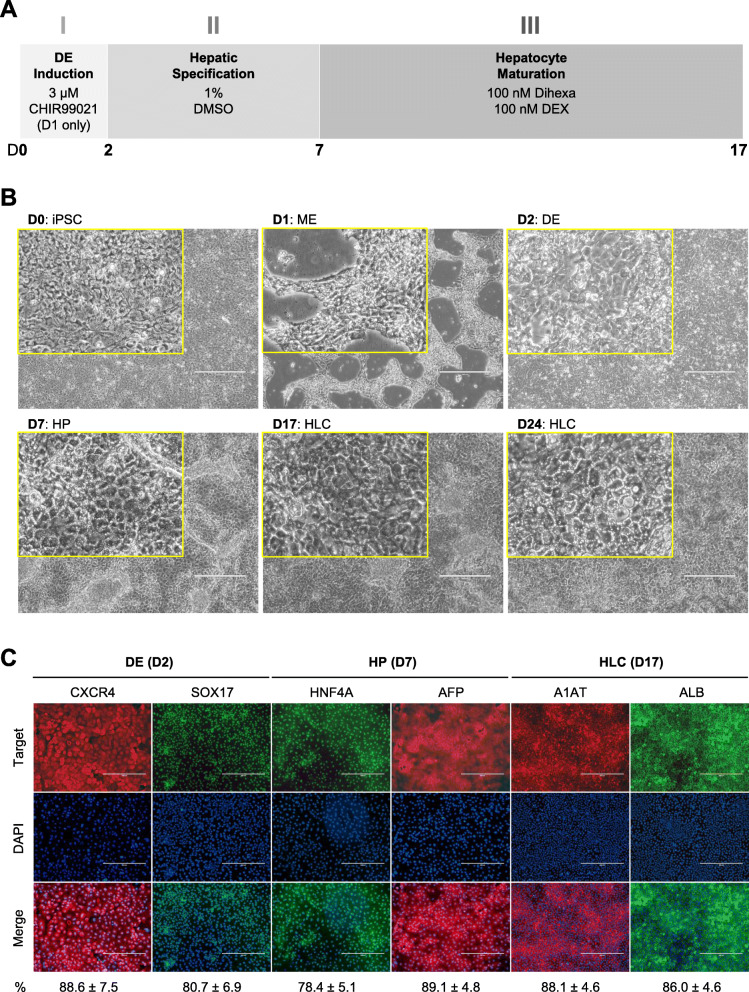
Small molecule-based protocol for the differentiation of hepatocytes from induced pluripotent stem cells. **a** Schematic diagram showing the three-phase differentiation process. **b** Representative images showing sequential morphological changes at each stage of the differentiation. iPSC, induced pluripotent stem cell; ME, mesendoderm; DE, definitive endoderm; HP, hepatic progenitor; HLC, hepatocyte-like cell. Magnification: × 10, Insert: × 40, Scale bar: 400 μm. **c** Representative immunocytochemistry images demonstrating the expression of key markers at each stage of the differentiation. The percentage of marker positive cells, presented as mean ± SD of three independent experiments, is listed at the bottom under each marker. Scale bar: 400 μm

HLCs were differentiated from iPSCs using a protocol that only involves SMs [12]. The protocol comprises of three phases (Fig. 1a). In the first phase, the cells were treated for 24 h with 3 μM CHIR99021, a potent and highly selective GSK-3β inhibitor [20], followed by 24 h of non-directed differentiation. Cells changed morphology from that of single-cell iPSC culture to dense, bright clusters at 24 h, which were presumably mesendoderm (ME) cells, followed by a pedal-like morphology at 48 h (Fig. 1b). Immunocytochemistry (ICC) staining showed expression of CXCR4 and SOX17 at 48 h, indicative of DE induction (Fig. 1c). In the second phase, the cells were treated with 1% DMSO for 5 days to specify a hepatic fate. The cells underwent a rapid change in morphology and a spurt of proliferation shortly after the treatment. At the end of the 5-day treatment, cells exhibited a typical hepatic progenitor (HP) morphology (Fig. 1b) and expressed HNF4A and AFP, as evidenced by ICC staining (Fig. 1c). In the final phase, cells were further treated for 10 days with dihexa, a potent and stable HGF receptor agonist [21], in combination with dexamethasone, a glucocorticoid mimetic, for hepatocyte maturation. On day 17, the cells displayed a typical cobblestone morphology resembling primary hepatocytes (Fig. 1b). These cells also demonstrated expression of hepatocyte markers A1AT and ALB in ICC staining (Fig. 1c). We extended the maturation stage for 7 more days. On day 24, the cells became more angular and displayed brighter junctures, suggesting cellular polarity and canaliculi formation (Fig. 1b).

### Hepatic functions of HLCs derived by SMs

**Fig. 2:**
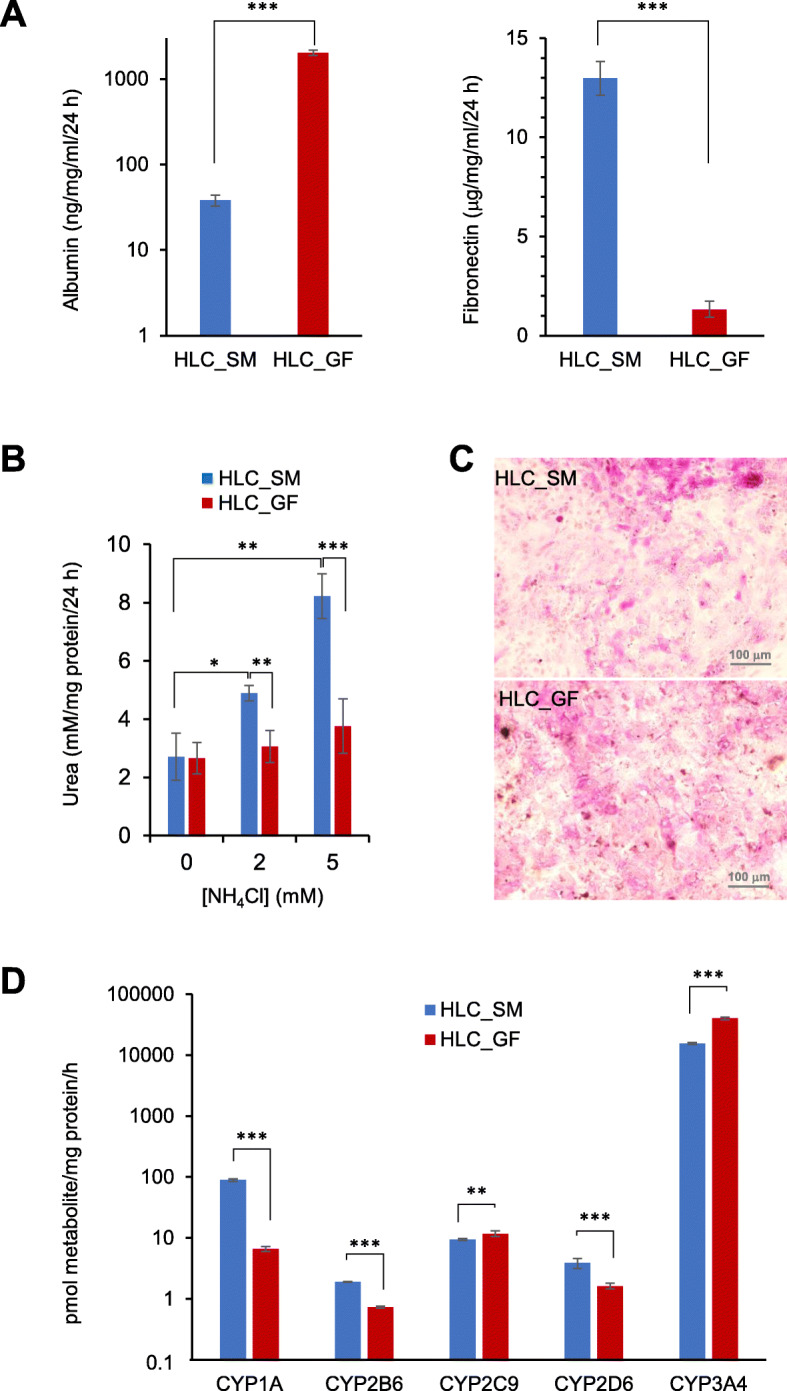
Functional analysis of hepatocyte-like cells derived using the small molecule-based protocol in comparison to those derived using the growth factor-based protocol. **a** Secretion of serum proteins (albumin and fibronectin). Wells without seeded cells were included as blank. **b** Urea synthesis in small molecule-derived hepatocyte-like cells upon challenging with ammonia. Wells without seeded cells were included as blank control. **c** Representative PAS staining images showing glycogen storage. **d** Basal level activity of major cytochrome P450 enzymes. HLC_SM, hepatocyte-like cells derived using small molecules; HLC_GF, hepatocyte-like cells derived using growth factors; CYP, cytochrome P450. All values are presented as mean ± SD. *n* = 4 for serum protein secretion, and *n* = 3 for urea synthesis and CYP activity. * *p* < 0.05, ** *p* < 0.01, *** *p* < 0.001 by unpaired two-tailed *t*-test

We next assessed whether HLC_SM possessed characteristic hepatic functions. One of the key functions of hepatocytes is the production of serum proteins. ELISA results showed that HLC_SM secreted both ALB and fibronectin into the medium at levels comparable to HLC_GF, with ALB higher for HLC_GF but fibronectin higher for HLC_SM (Fig. 2a). ALB is an adult hepatic marker while AFP is a typical fetal liver protein, and it is generally held that hepatocyte maturity is characterized by high levels of ALB expression and concomitant low levels of AFP expression. Therefore, we assessed the relative expression of ALB and AFP in HLC_SM in comparison to HLC_GF (Supplemental Fig. 1). Expression of either ALB or AFP in HLC_SM was slightly lower than that in HLC_GF, but the differences were statistically insignificant. In addition, ALB/AFP ratio was nearly identical for the two cell types (0.97 in HLC_SM vs. 0.99 in HLC_GF), suggesting that HLC_SM had similar maturity with HLC_GF.

Ureogenic capacity is another indicator of functional liver cell. HLC_SM showed increased synthesis and release of urea into the culture medium upon incubation with NH_4_Cl, with levels significantly higher than HLC_GF (Fig. 2b). Another important function of hepatocytes is glycogen storage. PAS staining of HLC_SM displayed extensive cytoplasmic staining (pink to purple) indicative of glycogen storage, at levels similar to those in HLC_GF (Fig. 2c).

Hepatocytes are capable of clearing xenobiotics via metabolism through cytochrome P450 (CYP) isoenzymes. Therefore, CYP activity is the most critical attribute of hepatocytes for toxicological applications. We used LC/MS to assess the basal activity of major CYP isoenzymes in HLC_SM. The result showed that HLC_SM had activity levels on par with HLC_GF for CYP1A, CYP2B6, CYP2C9, CYP2D6, and CYP3A (Fig. 2d). However, CYP2C19 activity was not detectable in either cell types.

### Genome-wide characterization of HLC_SM

To explore the molecular mechanisms underlying in vitro hepatocyte differentiation, as well as to obtain an unbiased assessment of the HLCs differentiated using SMs, we performed whole transcriptome gene expression analysis on cells at different stages of the SM-driven differentiation and also compared the final HLCs to PHHs, adult liver tissue, fetal liver tissue, HLC_GF, and commercially available HLCs. All RNA samples passed the RNA quality analysis with RNA integrity number (RIN) ≥ 8.0. Before data analysis, all arrays referred to in this study were assessed for data quality with all quality assessment metrics (including spike-in controls during target preparation and hybridization) within default boundaries. Three datasets downloaded from the GEO database named “HLC_Wruck” [18], “HLC_Yang_1,” and “HLC_Yang_2” were also included for comparison (reference datasets). Totally, 45 datasets were included in the study (Table 1).

**Table 1:** List of samples in the study

ID	Description	Replicate #
iPSC_1	Small molecule-driven differentiation on day 0 (iPSC)	1
iPSC_2	Small molecule-driven differentiation on day 0 (iPSC)	2
iPSC_3	Small molecule-driven differentiation on day 0 (iPSC)	3
DE_SM_1	Small molecule-driven differentiation on day 2 (definitive endoderm)	1
DE_SM_2	Small molecule-driven differentiation on day 2 (definitive endoderm)	2
DE_SM_3	Small molecule-driven differentiation on day 2 (definitive endoderm)	3
SM_D04_1	Small molecule-driven differentiation on day 4	1
SM_D04_2	Small molecule-driven differentiation on day 4	2
SM_D04_3	Small molecule-driven differentiation on day 4	3
HP_SM_1	Small molecule-driven differentiation on day 7 (hepatic progenitor)	1
HP_SM_2	Small molecule-driven differentiation on day 7 (hepatic progenitor)	2
HP_SM_3	Small molecule-driven differentiation on day 7 (hepatic progenitor)	3
SM_D12_1	Small molecule-driven differentiation on day 12	1
SM_D12_2	Small molecule-driven differentiation on day 12	2
SM_D12_3	Small molecule-driven differentiation on day 12	3
SM_D14_1	Small molecule-driven differentiation on day 14	1
SM_D14_2	Small molecule-driven differentiation on day 14	2
SM_D14_3	Small molecule-driven differentiation on day 14	3
HLC_SM_D17_1	Small molecule-driven differentiation on day 17 (hepatocyte-like cell)	1
HLC_SM_D17_2	Small molecule-driven differentiation on day 17 (hepatocyte-like cell)	2
HLC_SM_D17_3	Small molecule-driven differentiation on day 17 (hepatocyte-like cell)	3
HLC_SM_D24_1	Small molecule-driven differentiation on day 24 (hepatocyte-like cell)	1
HLC_SM_D24_2	Small molecule-driven differentiation on day 24 (hepatocyte-like cell)	2
HLC_SM_D24_3	Small molecule-driven differentiation on day 24 (hepatocyte-like cell)	3
DE_GF_1	Growth factor-driven differentiation on day 7 (definitive endoderm)	1
DE_GF_2	Growth factor-driven differentiation on day 7 (definitive endoderm)	2
DE_GF_3	Growth factor-driven differentiation on day 7 (definitive endoderm)	3
HP_GF _1	Growth factor-driven differentiation on day 14 (hepatic progenitor)	1
HP_GF_ 2	Growth factor-driven differentiation on day 14 (hepatic progenitor)	2
HP_GF_ 3	Growth factor-driven differentiation on day 14 (hepatic progenitor)	3
HLC_GF _1	Growth factor-driven differentiation on day 23 (hepatocyte-like cell)	1
HLC_GF _2	Growth factor-driven differentiation on day 23 (hepatocyte-like cell)	2
HLC_GF _3	Growth factor-driven differentiation on day 23 (hepatocyte-like cell)	3
HLC_iCell_1	iCell hepatocytes from FCDI	1
HLC_iCell_2	iCell hepatocytes from FCDI	2
Liver_A_1	Adult liver	1
Liver_A_2	Adult liver	2
Liver_F_1	Fetal liver	1
Liver_F_2	Fetal liver	2
PHH_1	Primary human hepatocytes	1
PHH_2	Primary human hepatocytes	2
PHH_3	Primary human hepatocytes	3
HLC_Wruck	Reference dataset (GSM3236134)	1
HLC_Yang_1	Reference dataset (GSM1633257)	1
HLC_Yang_2	Reference dataset (GSM1633258)	1

### Gene expression changes during the course of SM-driven hepatocyte differentiation

**Fig. 3:**
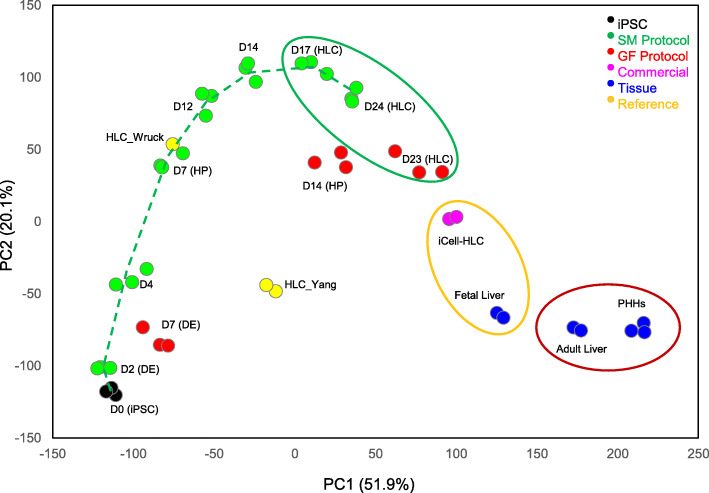
Principal component analysis (PCA) plot showing the differentiation track (dotted line) and similarities/dissimilarities among the samples. The two axes PC1 and PC2 represent the first two principal components identified by the analysis. The percentage contribution of each component to the overall source of variation is included in the parentheses following the component name. Color codes of different cell types are shown at the top-right corner. The timing of the small molecule-driven and growth factor-driven differentiation is denoted by D (day) followed by the number of the day. iPSC, induced pluripotent stem cell; DE, definitive endoderm; HP, hepatic progenitor; HLC, hepatocyte-like cell; PHHs, primary human hepatocytes. HLC_Wruck and HLC_Yang are reference datasets

PCA plot of all the 45 datasets is shown in Fig. 3. Starting from iPSCs (day 0), the differentiation proceeded progressively, through the DE (day 2) and HP (day 7) stages, and formed a differentiation track, finally arriving at the HLCs (day 17 and day 24). It is worth noting that the differentiation moved forward rapidly at the beginning (day 0–day 12) but slowed down afterwards, and HLCs on day 24 did not differ much from those of day 17. Also, cells at DE and HP stages of the GF-driven differentiation did not fall on the SM-driven differentiation track, suggesting the GF-driven differentiation may follow a different track. It was also noted that in many cases the three replicates of the SM-driven differentiation at each time-point were close to each other, while those of the GF-driven differentiation fell relatively apart, suggesting differentiation using SMs was more reproducible than that using GFs.

**Fig. 4:**
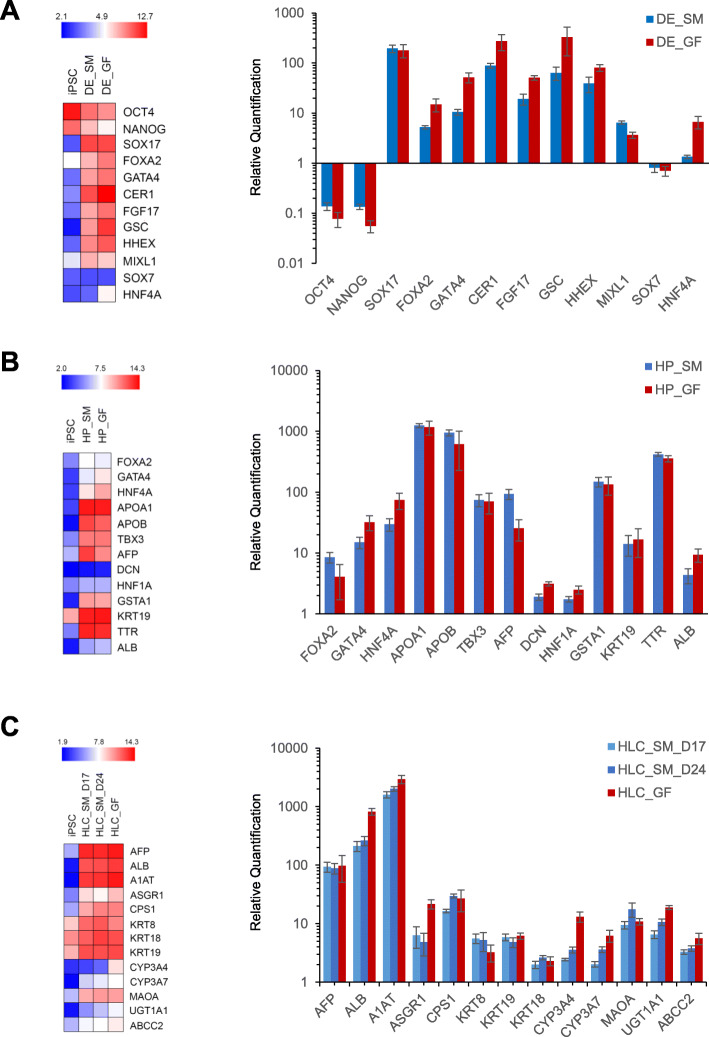
Expression of key marker genes at each stage of the hepatocyte differentiation. **a** Definitive endoderm markers. **b** Hepatic progenitor markers. **c** Hepatocyte markers. Left panel, heat maps. The expression data are the average of three replicates in log2 scale and color-coded as shown in the scheme at the top. Right panel, bar graphs. The expression values are relative to the starting induced pluripotent stem cells (average = 1). DE_SM, definitive endoderm derived using small molecules; DE_GF, definitive endoderm derived using growth factors; HP_SM, hepatic progenitor derived using small molecules; HP_GF, hepatic progenitor derived using growth factors; HLC_SM_D17, hepatocyte-like cells derived using small molecules at day 17; HLC_SM_D24, hepatocyte-like cells derived using small molecules at day 24; HLC_GF, hepatocyte-like cells derived using growth factors

Gene expression analysis showed that at the end of DE induction, cells exhibited marked decrease in the expression of pluripotent markers *OCT4* and *NANOG*, and much elevated expression of DE makers *SOX17*, *FOXA2*, *GATA4*, *CER1*, *FGF17*, *GSC*, *HHEX*, and *MIXL1*, to extents on par with DE cells derived using GFs (Fig. 4). To demonstrate that the SM differentiation did not result in embryonic endoderm formation, we also examined the expression of *SOX7* and *HNF4A* in the DE cells. Neither genes were elevated in SM-DE cells; in contrast, expression of *HNF4A* was increased in GF-DE cells.

Similarly, at the end of hepatic specification (day 7), a repertoire of HP markers was enormously expressed, including *FOXA2*, *GATA4*, *HNF4A*, *APOA1*, *APOB*, *TBX3*, *AFP*, *GSTA1*, *KRT19*, and *TTR*. Expression of *DCN* and *HNF1A* was also significantly higher than iPSCs, though to a lesser extent. It is also worth noting that elevated expression of *ALB* was observed as early as the HP stage. Expression of these markers in GF-derived HP cells were very similar.

A panel of hepatocyte markers was also highly expressed in the final HLCs, including *AFP*, *ALB*, *A1AT*, *ASGR1*, *CPS1*, *KRT8*, *KRT19*, *KRT18*, *CYP3A4*, *CYP3A7*, *MAOA*, *UGT1A1*, and *ABCC2*. In general, day 24 HLCs had slightly higher expression than day 17 HLCs; however, the difference was not significant in any case. Similarly, HLC_GF had slight but not significant higher expression than HLC_SM (day 17 and day 24) for most of the genes. It is interesting to note that both *CYP3A4* (the adult isoform of *CYP3A*) and *CYP3A7* (the fetal isoform) had higher expression in HLC_GF than in HLC_SM.

### Comparison of HLC_SM with PHHs and liver tissues

To obtain an unbiased assessment of the HLC_SM, we further compared these HLCs to PHHs, adult liver tissue, fetal liver tissue, commercially available HLCs (HLC_iCell), and HLC_GF. The microarray datasets were first analyzed by PCA to compare the different cell types. PCA provides a means to reduce high-dimensional gene expression data into few principal components [22]. Gene expression profiles are considered to be similar when samples fall in close proximity to one another in PCA plot. As shown in Fig. 3, apparently, HLC_SM_D17, HLC_SM_D24, and HLC_GF form one cluster, HLC_iCell and fetal liver form another one, and adult liver and primary hepatocytes form a third cluster. However, the reference datasets (HLC_Wruck, HLC_Yang) did not fall into any of the clusters.

Within the clusters, the three replicates of each cell type formed subclusters and separated from one another. Along the axis of the first principal component (PC1), the order of the cell groups, from left to right, was HLC_SM_D17, HLC_SM_D24, HLC_GF, HLC_iCell, fetal liver, adult liver, and PHHs.

**Fig. 5:**
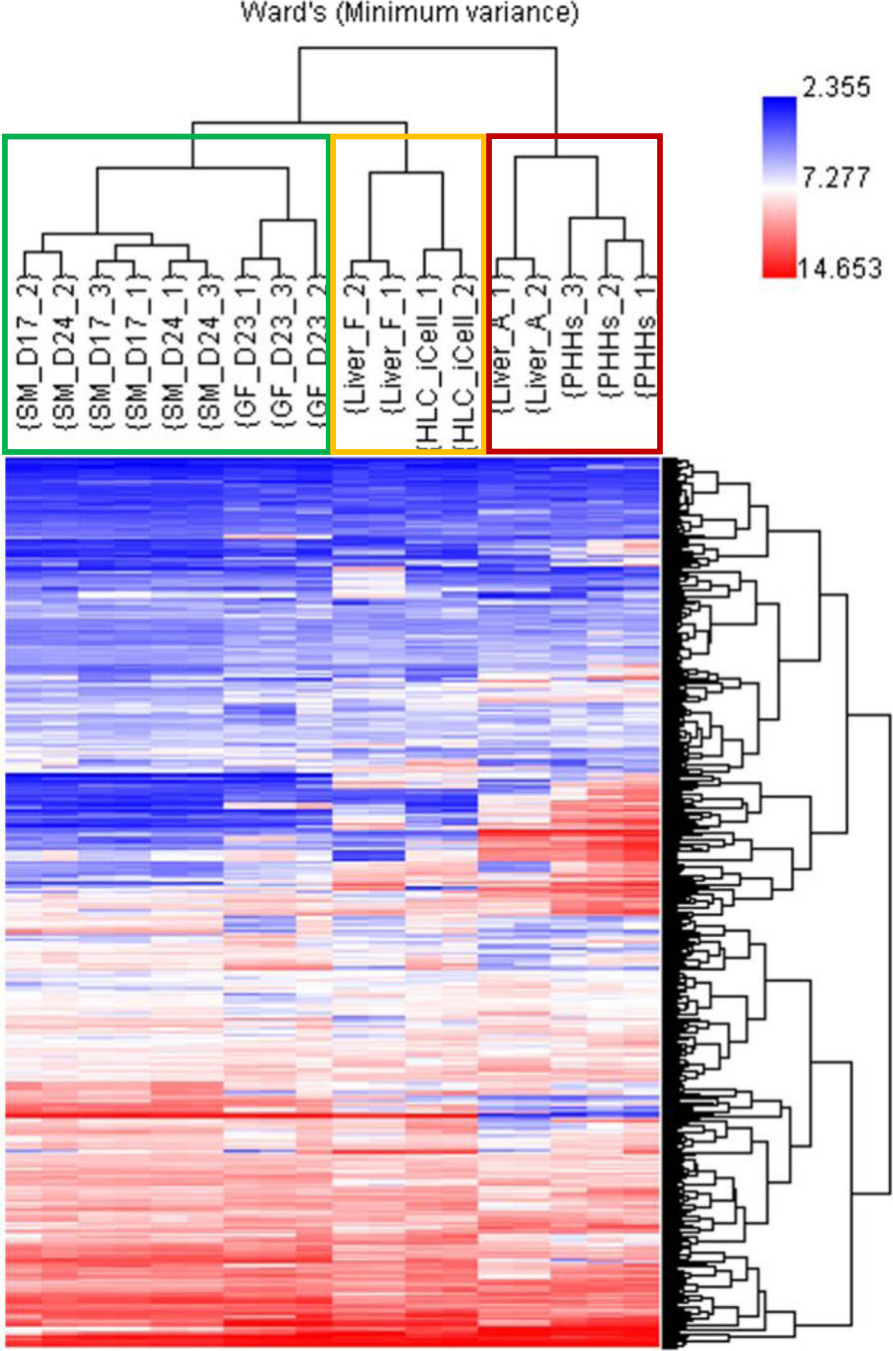
Hierarchical clustering analysis (HCA) of all the samples. The clustering was based on 520 probesets representing hepatotoxicity-related genes, drug-metabolizing enzymes, transporters, and nuclear receptors (see main text). The list of genes is provided in Supplemental Table 1. The clustering was performed through Ward’s minimum variance linkage on normalized expression data. The dendrogram on the right of the image shows clusters of genes (names not shown), while that on the top of the image shows clusters of samples with names shown. SM, small molecule; GF, growth factor; D, day of differentiation; Liver_F, fetal liver; Liver_A, adult liver; HLC_iCell, iCell hepatocyte-like cells (FCDI); PHHs, primary human hepatocytes

The datasets were further analyzed by hierarchical clustering analysis (HCA) based on 520 probesets [23] (Supplemental Table 1) representing hepatotoxicity (cholestasis, steatosis, phospholipidosis, non-genotoxic hepatocarcinogenicity, necrosis, and general hepatotoxicity) related genes [24], and genes related to drug-metabolizing enzymes (phases I and II), transporters, and nuclear receptors [25]. Using Ward’s minimum variance method [26], HCA yielded a hierarchy of clusters presented in the form of a dendrogram based on the similarities among samples. As shown in Fig. 5, the cell types were grouped into two major clusters: adult liver tissues and primary hepatocytes form one major cluster, and all other cells form another major cluster, within which HLC_SM and HLC_GF form one subcluster, and HLC_iCell form another subcluster with fetal liver.

### Expression of liver drug-metabolizing enzymes, transporters, and nuclear receptors

**Fig. 6:**
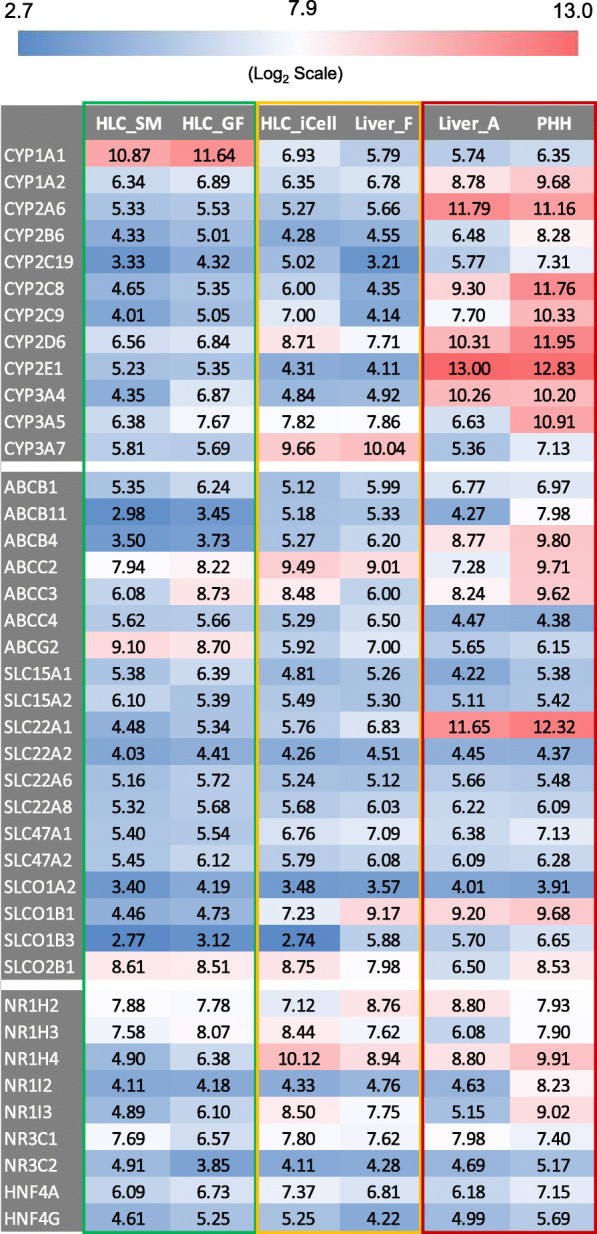
Relative gene expression of major cytochromes P450 enzymes (top), functional transporters (middle), and nuclear receptors (bottom). Gene expression value for each enzyme, transporter, or nuclear receptor was the average of the samples in each group and is in log2 scale and color-coded as shown in the scheme located at the top of the figure. HLC_SM, hepatocyte-like cells derived using small molecules; HLC_GF, hepatocyte-like cells derived using growth factors; HLC_iCell, iCell hepatocyte-like cells (FCDI); Liver_F, fetal liver; Liver_A, adult liver; PHHs, primary human hepatocytes

Expression of liver drug-metabolizing enzymes, transporters, and nuclear receptors are important attributes of hepatocytes for their toxicological applications. Relative gene expression of major phase I drug-metabolizing CYP enzymes and functionally relevant transporters and nuclear receptors were therefore compared across the different cell types. As shown in Fig. 6, there were enormous differences in the expression of these CYP enzymes among the cell types. Overall, PHHs and adult liver were very similar and had the highest expressions. HLC_SM and HLC_GF were alike and had the lowest expressions. HLC_iCell were on par with fetal liver and had expressions slightly higher than HLC_SM and HLC_GF. An exception is *CYP1A1*, which had much higher expression in HLC_SM and HLC_GF than in other cell types. The reason for this is unknown. Notably, *CYP3A7*, a fetal phenotype in the *CYP3A* family, was highly expressed in both HLC_iCell and fetal liver compared to other HLCs, PHHs, and adult liver. However, the adult phenotypes of the enzyme, *CYP3A4*, had much higher expression in PHHs and adult liver than in other cell types. In addition, expression of *CYP3A4* in HLC_GF was higher than in HLC_SM, and even higher than in HLC_iCell and fetal liver.

Expression of functionally relevant transporters and nuclear receptors (Fig. 6) generally followed the same pattern as the CYP enzymes, with PHHs and adult liver the highest, HLC_SM and HLC_GF the lowest, and HLC_iCell and fetal liver in between. Exceptions were *ABCG2* and *SLCO2B1*, which had higher expression in HLCs (_SM, _GF and _iCell) than in liver tissues and PHHs. Some other genes, *SLC22A2*, *SLCO1A2*, *NR3C2*, and *NHF4G*, had very similar expressions across all cell types.

### C/T classification of HLC_SM using CellNet

**Fig. 7:**
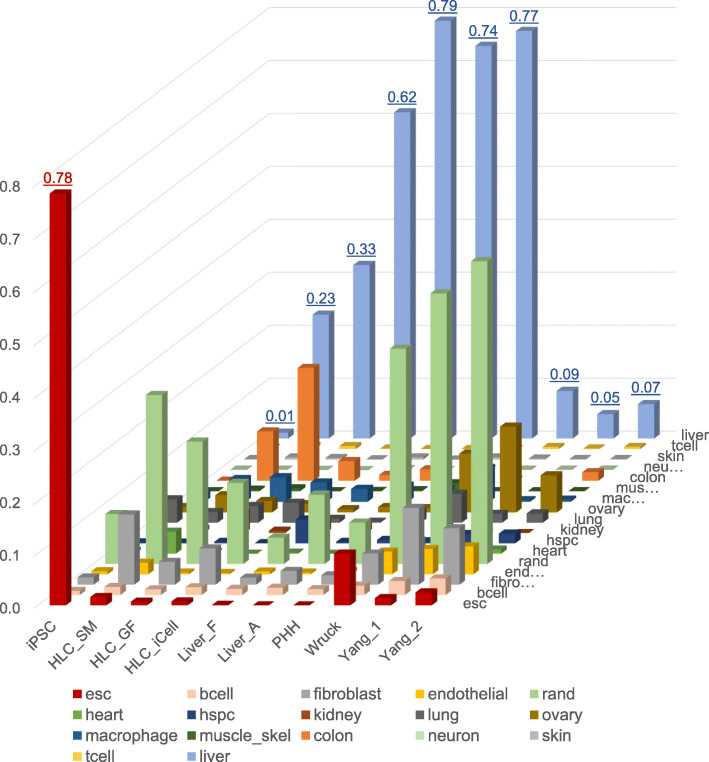
Cell and tissue type classification using CellNet. Liver classification average score for each sample is shown on top of the bar. The “esc” average score for the iPSC samples is also shown. iPSC, induced pluripotent stem cells. HLC_SM, hepatocyte-like cells derived using small molecules; HLC_GF, hepatocyte-like cells derived using growth factors; HLC_iCell, iCell hepatocyte-like cells (FCDI); Liver_F, fetal liver; Liver_A, adult liver; PHH, primary human hepatocytes. HLC_Wruck and HLC_Yang are reference datasets. esc, embryonic stem cell; bcell, B cell; rand, random (unknown); hspc, hematopoietic stem and progenitor cell; muscle-skel, musculoskeletal tissue; tcell, T cell

We further characterized the HLC_SM using CellNet, a bioinformatics platform quantifying how closely engineered cell populations resemble their target cell type [19]. Using the transcriptomic data, CellNet calculates the probability that query samples express C/T-specific gene regulatory network (GRN) genes to an extent that is indistinguishable from each C/T type in its training data set, namely, the C/T classification score [19]. As shown in Fig. 7, the starting iPSCs were classified exclusively as “esc” (embryonic stem cell) with an average score of 0.78. PHHs and liver tissues were classified exclusively as “liver” with scores between 0.74 and 0.79. However, all the HLCs showed mixed classifications majorly including “liver,” “colon,” “fibroblast,” and “rand” (random or unknown). For “liver” classification, HLC_iCell scored 0.62, HLC_GF 0.33, and HLC_SM 0.23. In comparison, the three reference HLCs scored below 0.1 for “liver” and had relatively higher classification scores for “esc”.

### Identification of pathways underlying differences between HLC_SM and HLC_GF

Thus far, we have shown that HLC_SM was similar to HLC_GF in terms of both overall gene expression and in the expression of hepatic markers; in addition, HLC_SM manifested key hepatic functions on par with HLC_GF. Nevertheless, differences between HLCs generated using these two differentiation methods were still evident. Comparison of global gene expression between HLC_SM and HLC_GF identified 3480 differentially expressed genes (DEGs) using a criterion of FC > 2.0 and *p* < 0.05 (Supplemental Table 2). Of these DEGs, 2302 were upregulated in HLC_SM compared to HLC_GF and 1178 downregulated.

The 3480 DEGs between HLC_SM and HLC_GF were subjected to pathway analysis using DAVID. A total of 43 KEGG pathways were enriched by the DEGs (Supplemental Table 3). Among them, 14 were found to be directly or indirectly involved in hepatocyte differentiation (Table 2). Apart from the *signaling pathways regulating pluripotency of stem cells*, two signal transduction pathways, *Wnt signaling pathway* and *TGF-beta signaling pathway*, both regulating stem cell differentiation and developmental processes, were overrepresented by the DEGs. In addition, eight pathways affecting cellular community (such as cell-cell and cell-matrix interactions) were also enriched by the DEGs, including *focal adhesion*, *ECM-receptor interaction*, *adherens junction*, *tight junction*, *gap junction*, *regulation of actin cytoskeleton*, *PI3K-Akt signaling pathway*, and *Hippo signaling pathway.* These pathways play important roles in stem cell differentiation thus affecting the quality of the resultant hepatocytes. Moreover, three pathways directly related to hepatocyte function, *bile secretion*, *insulin signaling pathway*, and *PPAR signaling pathway*, were also identified from the DEGs, suggesting functional differences between the HLC_SM and HLC_GF.

**Table 2:** KEGG pathways directly or indirectly involved in hepatocyte differentiation

Pathway	Count	***P*** value	Fold enrichment
*Pluripotency, differentiation, and development*			
Signaling pathways regulating pluripotency of stem cells	27	0.0572	1.42
Wnt signaling pathway	32	0.0027	1.71
TGF-beta signaling pathway	22	0.0034	1.93
*Cellular community*			
Focal adhesion	50	0.0000	1.79
ECM-receptor interaction	28	0.0000	2.37
Adherens junction	18	0.0124	1.87
Tight junction	21	0.0112	1.78
Gap junction	18	0.0815	1.50
Regulation of actin cytoskeleton	37	0.0795	1.30
PI3K-Akt signaling pathway	67	0.0019	1.43
Hippo signaling pathway	35	0.0017	1.71
*Hepatocyte function*			
Bile secretion	17	0.0203	1.81
Insulin signaling pathway	26	0.0778	1.39
PPAR signaling pathway	16	0.0324	1.76

### Identification of transcriptional regulators to improve the maturity of HLC_SM

**Fig. 8:**
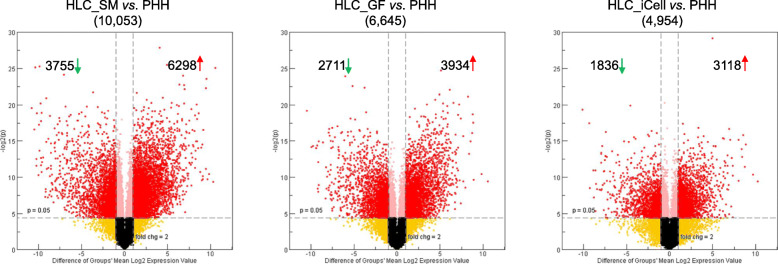
Volcano plots showing the differentially expressed genes (DEGs) between HLC_SM and PHH (left panel), between HLC_GF and PHH (middle panel), and between HLC_iCell and PHH (right panel). All DEGs are shown as red dots. Downregulated genes are on the top-left corners with numbers denoted by green arrows, and upregulated genes are on the top right corners with numbers denoted with red arrows. The total numbers of DEGs are included in the parentheses under the title

To explore transcriptional mechanisms governing gene expressions during HLC differentiation, we further calculated the DEGs between HLCs and PHHs. There were 4954 DEGs (Supplemental Table 4) identified in HLC_iCell (FC > 2.0 and *p* < 0.05), 6645 DEGs (Supplemental Table 5) in HLC_GF, and 10,053 DEGs (Supplemental Table 6) in HLC_SM (Fig. 8).

**Fig. 9:**
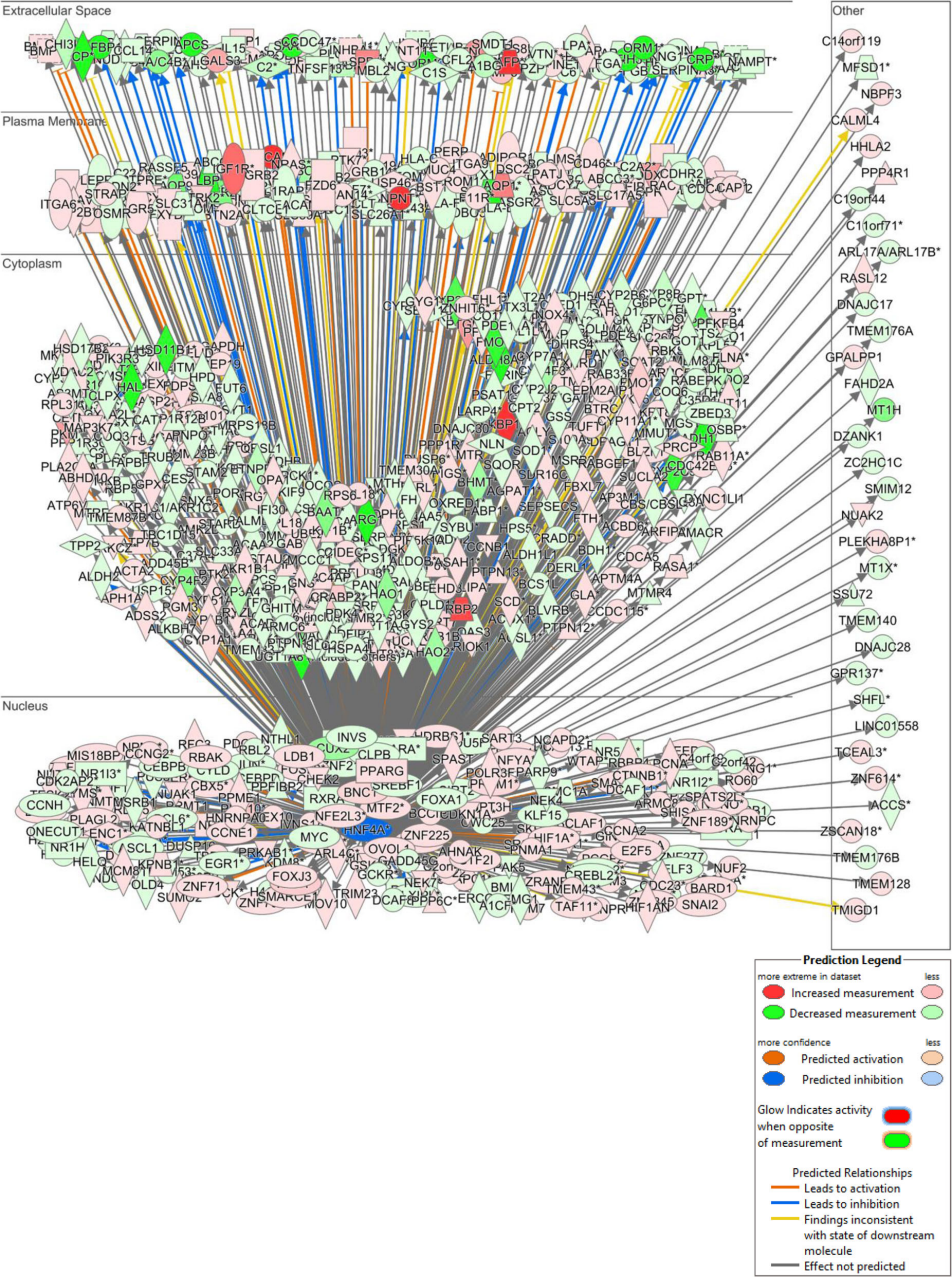
A gene network in Ingenuity Pathway Analysis illustrating the large number of downstream differentially expressed genes (DEGs) under the regulation of HNF4A. The DEGs are arranged in a subcellular layout and color-coded by expression changes as shown in the legend. The predicted action (activation or inhibition) and relationship for each DEG are also color-coded as shown in the legend

The 10,053 DEGs between HLC_SM and PHHs were further analyzed by Ingenuity Pathway Analysis focusing on upstream analysis to identify upstream regulators that may be responsible for the differential gene expression between HLCs and PHHs. We found 26 categories of upstream regulators (Table 3) with a total number of 2958 (Supplemental Table 7). We focused on the transcription regulators as they may be directly accountable for the differential gene expressions. A total of 480 transcription regulators were identified (Supplemental Table 8), 128 were with predicted action (either activation or inhibition), and 52 were among the DEGs (Table 4), with 13 of them each regulating more than 100 target molecules (DEGs). On top of the list is *HNF4A*, which regulated 750 DEGs in the dataset (Supplemental Table 9). The interactions of *HNF4A* with its downstream target genes are illustrated in Fig. 9. These target genes are localized in all subcellular locations of the cell, including 191 in the nucleus, 333 in cytoplasm, 106 on plasma membrane, and 86 in the extracellular space. These regulated genes belong to a variety of functional families, with 8 being cytokines, 221 enzymes, 10 G-protein coupled receptors, 10 growth factors, 3 ion channels, 42 kinases, 12 ligand-dependent nuclear receptors, 27 peptidases, 19 phosphatases, 82 transcription regulators, 1 translation regulator, 17 transmembrane receptors, and 71 transporters. Many of these target genes have predicted action (activation or inhibition) by *HNF4A*, with 55 predicted to be activated and 130 inhibited.

**Table 3:** List of 26 categories of upstream regulators identified for DEGs

	#	Predicted activation	Predicted inhibition
Biologic drug	33	1	5
Chemical - endogenous	230	11	17
Chemical - kinase inhibitor	43	0	10
Chemical - protease inhibitor	14	2	0
Chemical - other	6	0	0
Chemical drug	423	27	80
Chemical reagent	104	7	15
Chemical toxicant	108	18	8
Complex	70	13	9
Cytokine	56	11	6
Enzyme	258	28	25
Fusion gene/product	16	3	3
G-protein coupled receptor	41	7	1
Group	113	16	11
Growth factor	64	29	2
Ion channel	10	1	1
Kinase	150	28	8
Ligand-dependent nuclear receptor	35	4	5
MicroRNA	110	0	47
Peptidase	50	10	1
Phosphatase	23	2	0
Transcription regulator	480	78	50
Translation regulator	16	3	0
Transmembrane receptor	56	7	3
Transporter	63	5	5
Other	386	53	26
**Total**	**2958**	**364**	**338**

**Table 4:** List of 52 upstream transcription factors within the DEGs

Transcription regulator	Fold change	Predicted activation	Activation ***z***-score	***P*** value of overlap	# Target molecules in dataset
HNF4A	− 3.385	Inhibited	− 5.745	2.70E−27	750
TP53	− 2.129	Activated	3.271	7.29E−48	726
MYC	− 2.262	Activated	5.269	6.14E−33	486
CTNNB1	5.332	Activated	4.818	3.26E−28	384
SMARCA4	2.68	Activated	2.141	1.39E−18	256
STAT3	− 3.854	Activated	2.077	6.50E−14	236
HNF1A	− 2.214	Inhibited	− 4.778	5.03E−20	213
HIF1A	6.735	Activated	4.443	1.26E−13	176
PPARGC1A	47.619	Inhibited	− 5.494	1.69E−19	168
POU5F1	− 4.011	Activated	3.538	1.80E−06	123
YAP1	4.979	Activated	3.935	1.89E−11	117
SMAD3	2.214	Activated	3.208	5.88E−11	116
KLF3	− 2.047	Inhibited	− 2.844	4.20E−05	115
NOTCH1	2.518	Activated	2.574	9.65E−06	98
MITF	2.081	Activated	3.233	3.52E−05	87
ETS1	19.431	Activated	2.415	1.34E−05	84
TWIST1	6.797	Activated	2.226	1.63E−10	79
SOX4	50.964	Activated	3.166	2.79E−06	75
SMAD7	4.099	Inhibited	− 2.615	1.64E−10	74
MYB	− 2.015	Activated	3.237	2.10E−05	69
EGR1	− 6.18	Activated	2.985	1.19E−03	66
LEF1	11.741	Activated	2.657	1.37E−05	61
CBX5	3.806	Inhibited	− 3.141	3.15E−09	59
RBPJ	2.248	Inhibited	− 2.082	1.14E−04	59
NFAT5	2.319	Activated	2.291	2.97E−07	48
MLXIPL	− 6.246	Activated	4.068	4.44E−04	47
TEAD1	2.73	Activated	3.536	3.29E−08	39
IRF7	− 4.895	Inhibited	− 3.731	1.00E+00	39
MTPN	2.647	Activated	3.39	1.23E−04	36
KLF5	15.642	Activated	3.404	4.43E−06	31
HAND2	14.283	Activated	2.342	2.90E−04	30
PLAG1	18.451	Activated	2.835	2.52E−04	26
KLF15	12.019	Inhibited	− 3.222	4.42E−04	23
E2F6	2.874	Inhibited	− 2.32	3.98E−02	22
NFATC4	6.717	Activated	2.088	7.85E−03	15
EED	4.823	Inhibited	− 2.121	1.59E−01	14
ETV1	− 3.182	Activated	2.085	4.82E−04	14
CREB3L3	− 9.434	Inhibited	− 2.977	4.82E−04	14
SMARCD3	3.952	Activated	2.891	1.69E−04	13
MTDH	− 2.891	Activated	2.026	1.06E−01	13
BTG2	7.736	Inhibited	− 2.109	2.82E−02	11
NFIX	− 5.255	Inhibited	− 2.159	9.42E−03	11
CREBZF	2.133	Activated	3.105	8.44E−03	10
STAT2	− 2.78	Inhibited	− 2.38	1.00E+00	10
GLIS2	10.34	Inhibited	− 2.333	1.85E−02	9
PKNOX1	3.15	Activated	2.408	3.92E−02	9
NLRC5	− 6.873	Inhibited	− 2.053	2.50E−01	7
HIF3A	10.89	Inhibited	− 2.415	7.79E−02	6
JARID2	5.327	Inhibited	− 2.176	7.79E−02	6
SERTAD2	3.181	Activated	2.387	1.45E−01	6
ZFPM2	5.634	Activated	2.236	3.64E−01	5
TRIM25	− 2.353	Inhibited	− 2.195	1.12E−01	5

## Discussion

In this study, we evaluated a previously published protocol for hepatocyte differentiation from iPSCs that only involves SMs [12]. Using whole genome microarrays, we characterized gene expression changes during the course of the SM-driven differentiation (Fig. 3) and assessed the expression of some key marker genes in major stages of hepatocyte differentiation (Fig. 4). Increased expressions of key markers for DE (*CER1, FOXA2, GATA4, GSC, HHEX, MIXL1, SOX17*), HP (*AFP, FOXA2, GATA4, HNF4A, TBX3, TTR*) and hepatocyte (*A1AT, AFP, ALB, ASGR1, CYP3A4*) were on the same magnitude as those reported by Siller et al. [12] assessed using qPCR. In addition, HLC differentiation efficiency, as assessed by immunocytochemistry and expressed as the percentage of A1AT positive cells, reached 88.1 ± 4.6%, compared to 90.1 ± 1.4%, 92.7 ± 2.9%, 88.7 ± 4.6% for the three iPSC lines (RA, RB, and RC), respectively, obtained by Siller et al. [12]. These results indicate that in the current study, we were able to successfully reproduce the SM-based hepatocyte differentiation protocol [12].

Significant progress has been made over the past decade on hepatocyte differentiation from human iPSCs, and consequently, a variety of protocols have been developed [27–30]. However, it is often difficult to evaluate and compare these protocols in an unbiased manner. Usually, the quality of the resultant cells was assessed in individual laboratories based on the expression of a selected set of markers, accompanied by limited functional analyses such as ALB secretion, glycogen production, ammonia uptake, and CYP enzyme activity assays [9], which are oftentimes qualitative or semi-quantitative and therefore are subject to biases from individual researchers. In the current study, we used whole genome transcription analysis combined with GRN analysis to characterize iPSC-derived HLCs. The method presented herein provides an unbiased assessment of HLC differentiation, and could be used to evaluate past differentiation results, improve current differentiation protocols, and guide future differentiation endeavors. In this regard, it is worth noting that three previously reported HLC preparations, which were used as reference materials in the current study, showed very low liver C/T classification scores (Fig. 7). Transcriptomic data showed these cells were at the early stages (close to HP) of hepatocyte differentiation (Fig. 3).

It should be pointed out that although only one iPSC line (S06) was used, the findings we presented in the current study are applicable to other iPSC lines. For iPSC-derived HLCs, inter-cell-line differences in global gene expression would arise mainly from two sources: (1) varying differentiation efficiency among cell lines for the protocol used; (2) genetic polymorphism among individuals. Regarding differentiation efficiency, the robustness of the SM-based protocol for the differentiation of different cell lines was demonstrated by Siller et al. [12]. The authors obtained very similar efficiencies among two ESC lines and three iPSC lines under the same differentiation condition. As for genetic polymorphism, a previous study [23] showed that differences in global gene expression among six PHH lines and among three hepatoma cell lines, respectively, are much smaller or negligible in comparison to the differences between the two cell types (PHH vs. hepatoma cell line). Similarly, in the current study, the inter-individual differences among the three PHH lines were dwarfed in comparison to the large discrepancies between HLC_SM and PHH (Fig. 3), further suggesting that genetic polymorphism among individuals was insignificant. Therefore, it is expected that the substantial differences observed in the current study between the different cell types (HLC_SM, HLC_GF, PHH) with the iPSC line used (S06) would be largely reproducible in other iPSC lines.

Collectively, the data presented in this study demonstrated that the quality of HLC_SM was on par with that of HLC_GF, in terms of both overall gene expression (Figs. 3 and 5) and the expression of metabolic enzymes, transporters, and nuclear receptors (Fig. 6). CellNet produced similar liver C/T classification scores for both cell preparations (Fig. 7). In addition, the two methods showed similar key marker expression at major stages of the differentiation (Fig. 4). More importantly, HLC_SM manifested key hepatic functions including serum protein secretion, urea release, glycogen storage, and CYP enzyme activity (Fig. 2). For some functions, such as ALB secretion, HLC_SM was inferior to HLC_GF; while for some other functions, such as fibronectin secretion, HLC_SM was superior to HLC_GF. Overall, however, the performance of these two cell types was on par with each other. Stem cell differentiation using SMs offers many advantages over GF-based differentiation methods, including cell permeability, reversibility, structural and functional versatility, and finetunability [11], and especially in terms of lower cost, higher reproducibility, and better safety. Although still in their infancy, it is anticipated that, with further improvements, SM-based protocols will become the mainstream in the field of hepatocyte differentiation in the foreseeable future. Nevertheless, there existed apparent differences between HLC_SM and HLC_GF in gene expression and in functional phenotypes. The KEGG pathways overrepresented by the DEGs between HLC_SM and HLC_GF (Table 2) could be further explored to find clues for further optimizing the SM-based differentiation protocol.

Transcriptomic characterization of the intermediate cells collected along the course of the differentiation revealed a dynamic nature of the process. On the PCA plot (Fig. 3), cell fate changed gradually from iPSC, through DE and HP, and reached HLC, forming a differentiation track. Changes in the transcriptome space were rapid at the beginning (up to day 12) but slowed down significantly thereafter. It is interesting to note that HLCs on day 24 did not change much from those of day 17, suggesting that further prolongation of the differentiation would not improve hepatocyte maturation. This is in accordance with the findings of a recent study by Raju et al. [31]. The authors carried out a meta-analysis of cross-species transcriptome data of in vitro HLC differentiation and in vivo mouse embryonic liver development, and found that HLCs cease further maturation at an equivalent stage of mouse embryonic day E13–15. Regardless of the species origin, or the protocols used for differentiation, HLCs appear to encounter universal roadblocks preventing their maturation. The reason for the “blocks” is not clear, but is likely due to some discordantly expressed genes between HLC differentiation and mouse live embryo development identified by the study, and in particular some mis-expressed transcription factors [31].

In line with the above findings by Raju et al. [31], the current study identified 52 transcription factors that may be held accountable for the global gene expression differences between HLC_SM and PHHs. For instance, *HNF4A* regulated 750 genes in HLC_SM that were differentially expressed compared with PHHs. Previous studies found that HNF4A plays a key role in liver development as it is essential for the expression of many mature hepatic proteins and the development of normal liver morphology [32]. Recombinant adenoviral vectors expressing HNF4A have been shown to improve HLC differentiation [33]. Therefore, it is reasonable to assume that through forced expression or knockdown of these transcription factors, either alone or in combination, HLC differentiation and maturation could be further improved. However, it should be borne in mind that for ectopic expression of growth factors, temporal control of the expression is critical. For instance, studies have shown that during liver organogenesis, HNF4A is initially expressed at the hepatic specification stage and only detectable as early as in hepatoblasts [34, 35]. Expression of HNF4A earlier, at the definitive endoderm stage, indicates production of embryonic rather than definitive endoderm (Schwartz et al., 2014). In consistence with these findings, the current study showed very low HNF4A expression at the DE stage in the SM-driven differentiation (Fig. 4a).

C/T classification using CellNet showed that HLCs differentiated either by SMs or GFs were a mixed population of cells with characteristic GRNs of liver, colon, fibroblast, and other unknown tissues. GRN analysis of HLC preparations established by three independent laboratories resulted in very similar conclusions [36]. In addition, our data also clearly pointed to the notion that HLCs were closer to fetal than adult hepatocytes, which is in accordance with a previous proteomics study [37]. Therefore, there are still vast differences between HLCs and primary hepatocytes. Indeed, the present study identified several thousand DEGs between HLCs and PHHs. Very similar findings were reported by Godoy et al. [36]. Hence, more effort is needed to further improve the maturation of HLCs. Inarguably, attaining maturation of HLCs comparable to that of PHHs is still a formidable challenge associated hepatocyte differentiation. Over the past years, many approaches have been explored to enhance maturation of HLCs, including the formation of tissue-like three-dimensional structure, coculture of HLCs and endothelial cells, the addition of SMs uncovered by screening, and overexpression of certain transcription factors [38]. Nevertheless, improvements on HLC maturation attained by these methods are very limited. A better understanding of the molecular and cellular basis in organogenesis of the liver coupled with emerging innovative technologies, such as 3-dimensional bioprinting, organ-on-chip microfluidics, and genome modification, may lead to breakthroughs in the field.

## Conclusions

In summary, the current transcriptomic study demonstrated the usefulness of the SM-based hepatocyte differentiation method through showing that HLCs differentiated using SMs were similar to those differentiated using GFs in global gene expression and in the expression of genes related to hepatotoxicity, drug-metabolizing enzymes, transporters, and nuclear receptors. However, there are still vast differences between HLCs differentiated using current protocols and primary hepatocytes, and therefore more effort is needed to further improve the maturation of HLCs. Our results offered new insights into the molecular basis of hepatogenesis and associated gene regulation and suggested ways for further improvements in hepatocyte differentiation in order to obtain more mature HLCs that could be used in toxicological studies.

## Supplementary information

**Additional file 1.** Supplemental Fig. 1. Comparison of albumin (ALB) and alpha-fetoprotein (AFP) expression between HLC_SM and HLC_GF. (PPTX 11227 kb)

**Additional file 2.** Supplemental Table 1. List of genes related to hepatotoxicity, drug-metabolizing enzymes, transporters, and nuclear receptors.

**Additional file 3 **Supplemental Table 2. List 3480 DEGs between HLC_SM and HLC_GF (FC > 2.0 and *p* < 0.05).

**Additional file 4.** Supplemental Table 3. List of 43 KEGG pathways enriched by the DEGs between HLC_SM and HLC_GF.

**Additional file 5.** Supplemental Table 4. List of 4954 DEGs between HLC_iCell and PHHs (FC > 2.0 and p < 0.05).

**Additional file 6.** Supplemental Table 5. List of 6645 DEGs between HLC_GF and PHHs (FC > 2.0 and p < 0.05).

**Additional file 7.** Supplemental Table 6. List of 10,053 DEGs between HLC_SM and PHHs (FC > 2.0 and p < 0.05).

**Additional file 8.** Supplemental Table 7. List of 2958 upstream regulators identified for the 10,053 DEGs between HLC_SM and PHHs.

**Additional file 9.** Supplemental Table 8. List of 480 transcription regulators identified for the 10,053 DEGs between HLC_SM and PHHs.

**Additional file 10 **Supplemental Table 9. List of the 750 DEGs in the dataset regulated by *HNF4A*.

## Data Availability

The datasets used and/or analyzed during the current study are available from the corresponding author on reasonable request. All data generated or analyzed during this study are included in this published article and its supplementary information files. Original microarray data will be deposited in the Gene Expression Omnibus (GEO) database.

## Abbreviations

A1AT: Alpha-1-antitrypsin
ALB: Albumin
AFP: Alpha-fetoprotein
ANOVA: Analysis of variance
BMP4: Bone morphogenetic protein 4
cDNA: Complimentary deoxyribonucleic acid
cRNA: Complementary ribonucleic acid
C/T: Cell and tissue
CXCR4: C-X-C chemokine receptor type 4
CYP: Cytochrome P450
DAVID: Database for Annotation, Visualization, and Integrated Discovery
DE: Definitive endoderm
DEG: Differentially expressed gene
dihexa: N-hexanoic-Tyr, Ile- (6) amino-hexanoic amide
DMEM: Dulbecco’s modified Eagle’s medium
DMSO: Dimethyl sulfoxide
ELISA: Enzyme-linked immunosorbent assay
FC: Fold change
FCDI: Fujifilm Cellular Dynamics, Inc.
FDR: False discovery rate
FGF2: Fibroblast growth factor 2
GF: Growth factor
GRN: Gene regulatory network
GSK: Glycogen synthase kinase
HCA: Hierarchical clustering analysis
HGF: Hepatocyte growth factor
HLC: Hepatocyte-like cell
HNF4A: Hepatocyte nuclear factor 4 alpha
HP: Hepatic progenitor
ICC: Immunocytochemistry
iDILI: Idiosyncratic drug-induced liver injury
IPA: Ingenuity Pathway Analysis
iPSC: Induced pluripotent stem cell
ITS: Insulin-transferrin-selenium
IVT: In vitro transcription
KEGG: Kyoto Encyclopedia of Genes and Genomes
MEM: Minimal essential medium
PAS: Periodic acid-Schiff
PCA: Principal component analysis
PHH: Primary human hepatocyte
RIN: RNA integrity number
RMA: Robust multi-array average
RPMI: Roswell Park Memorial Institute (medium)
SD: Standard deviation
SM: Small molecule
SOX17: SRY (sex-determining region Y)-box 17
