# Supplementary material for: Hepatocyte-like cells derived from human induced pluripotent stem cells using small molecules: implications of a transcriptomic study
Source: Stem Cell Res Ther. 2020 Sep 11;11:393. doi: 10.1186/s13287-020-01914-1 (PMC7488531; doi:10.1186/s13287-020-01914-1)
Supplement: Supplementary file 1 — Additional file 1. Supplemental Fig. 1. Comparison of albumin (ALB) and alpha-fetoprotein (AFP) expression between HLC_SM and HLC_GF. (PPTX 11227 kb) [file 13287_2020_1914_MOESM1_ESM.pptx]

## Slide 1
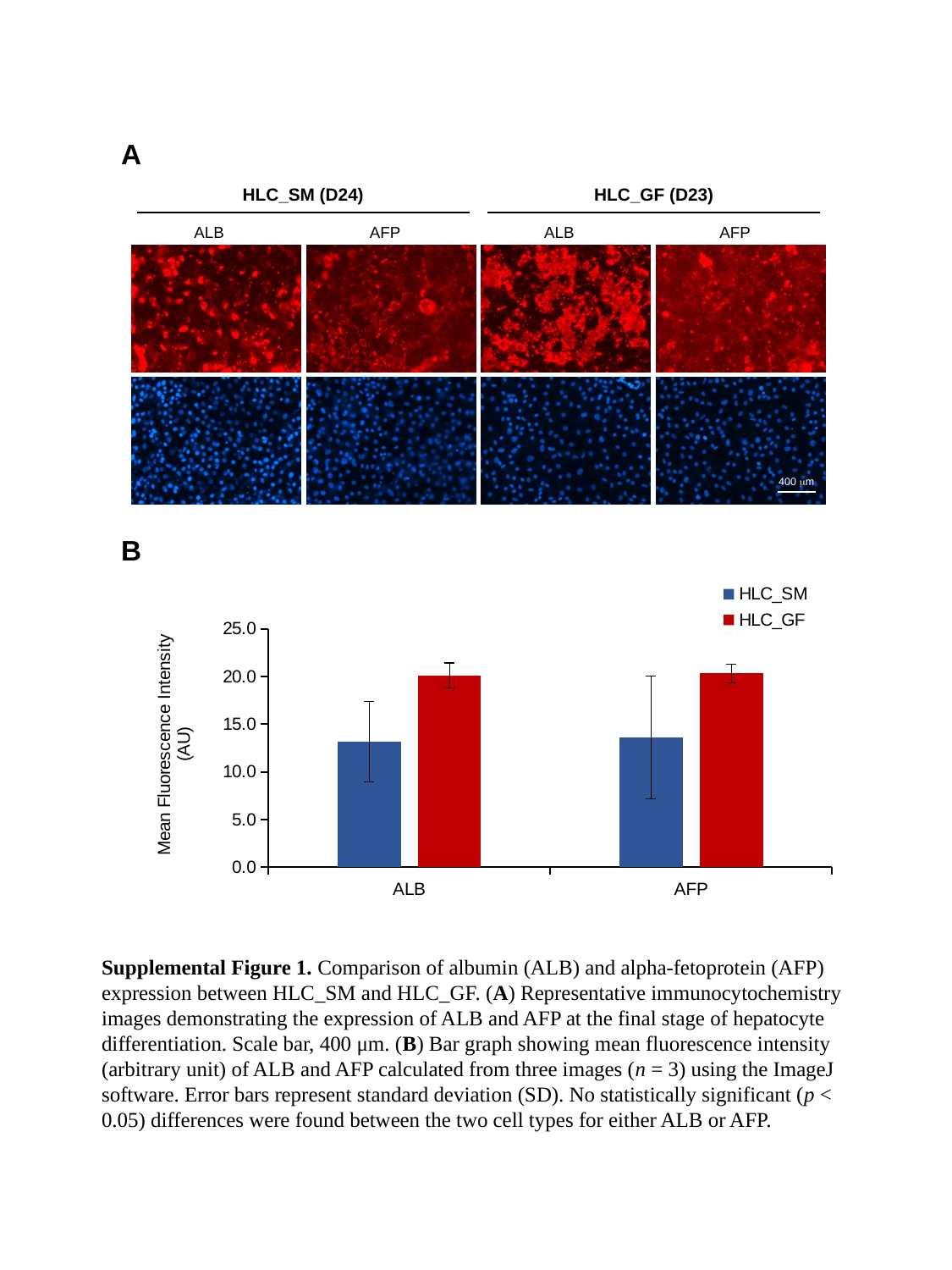

A
HLC_SM (D24)
HLC_GF (D23)
ALB
AFP
ALB
AFP
400 μm
B
### Chart
| Category | | |
|---|---|---|
| ALB | 13.151333333333334 | 20.122666666666664 |
| AFP | 13.609333333333334 | 20.324666666666666 |Supplemental Figure 1. Comparison of albumin (ALB) and alpha-fetoprotein (AFP) expression between HLC_SM and HLC_GF. (A) Representative immunocytochemistry images demonstrating the expression of ALB and AFP at the final stage of hepatocyte differentiation. Scale bar, 400 μm. (B) Bar graph showing mean fluorescence intensity (arbitrary unit) of ALB and AFP calculated from three images (n = 3) using the ImageJ software. Error bars represent standard deviation (SD). No statistically significant (p < 0.05) differences were found between the two cell types for either ALB or AFP.
